# Proposals for integrated public management of the menopausal transition and postmenopause through Spanish women's experiences: a qualitative study

**DOI:** 10.3389/frph.2024.1483267

**Published:** 2024-12-16

**Authors:** Clara Selva

**Affiliations:** Universitat Oberta de Catalunya (Estudis de Psicologia i Ciències de l'Educació - EPCE), Barcelona, Spain

**Keywords:** menopausal transition, postmenopause, menopause management, public health policy, women's health, qualitative research, governmental proposals, gender equity

## Abstract

**Introduction:**

In Spain, legislation protecting women during the menopausal transition and postmenopause is still in its early stages. For public policies to be effective, it is essential that their design, implementation, and evaluation actively involve women going through this stage. Only from their experience and perspective can the impact of measures be maximized and ensure that they truly respond to their needs and realities. The goal of this article is to identify and analyze proposals for action that public organizations in Spain could undertake to improve the management of and transition through menopause. On the basis of the initiatives identified through women's narratives, this study aims to establish a solid basis for the inclusion of future governmental policies and practices in Spain. Adapting to the economic conditions and sociocultural aspects specific to each region can stimulate reflection and debate on their policies regarding menopause.

**Methods:**

This qualitative study, which was conducted in Spain, was based on 20 semistructured interviews with women who had experienced physiological and symptomatic menopause in the last five years, were postmenopausal, and were actively employed. The interviews were audio-recorded, transcribed, and analyzed following an inductive thematic analysis.

**Results:**

The stories of the interviewees indicate three categories of government action comprising a set of specific measures. More specifically, the categories relate to: (a) proposals for accompaniment and social awareness, (b) educational and training proposals, and (c) political and regulatory proposals.

**Conclusions:**

This study identifies governmental proposals and specific actions to improve support for the menopausal transition and postmenopause in Spain. While its findings may be applicable to other countries, further research is needed to explore how they could be adapted to the different socioeconomic and cultural realities of those contexts. Ultimately, the proposals presented lay the groundwork for developing public policies and laws that respond to the needs of women, improving their well-being and promoting gender equity in public health.

## 1 Introduction

Menopause is a biological event that marks the end of menstruation as a result of the loss of ovarian function (1). The menopausal transition is influenced by age, health and sociocultural context (2) and is characterized by irregular periods and hormonal fluctuations. The phase after the end of the menstrual period is known as postmenopause (3).

Despite being a natural part of aging, menopause presents numerous challenges for women (4, 5). Many, particularly those under 40 years of age and those with limited resources, lack adequate information about this stage (6), hindering their preparation for its physical and emotional effects and impacting their well-being and quality of life (7). Furthermore, menopause is often misunderstood and mismanaged due to persistent gender biases in research funding, leading to a limited understanding of the condition beyond its physiological symptoms (8, 9). Crucially, sociocultural, educational, and environmental factors, which are vital for developing holistic support strategies, are often neglected (10, 11).

This lack of comprehensive understanding is reflected in public health policies that often fail to address the diverse needs of women experiencing menopause (12, 13). Healthcare systems, while striving for inclusion, may still perpetuate a medicalized view of menopause, focusing on standardization and neglecting individual experiences (14). This disconnect between needs and available support is further complicated by the sociocultural context (15), which can perpetuate myths, stigmas, and even discrimination associated with menopause and aging (16, 17).

The impact of menopause extends beyond individual well-being, significantly affecting women in the workplace (18). With a growing number of women over 50 remaining in the workforce (19), studies show that over 65% experience decreased productivity and efficiency due to menopausal symptoms, including difficulty concentrating, increased stress, and mood swings, which are often exacerbated by unsupportive work environments (20–22). The lack of inclusive policies and persistent stigma (23–25) contribute to absenteeism, reduced work hours, and even job loss (26, 27). Despite its recognition as a public health issue, menopause remains largely neglected in workplace management, with negative consequences for women's income, well-being, and overall quality of life (28).

While recent social and political initiatives have aimed to raise awareness and improve menopause management in the workplace (29–32), their limited scope and context-specific nature highlight the need for comprehensive and universal approaches (3). This gap in research and policy underscores the urgency of understanding the lived experiences of women navigating menopause and translating those experiences into effective support systems.

In Spain, despite a universal healthcare system and progressive gender equality policies, menopause remains neglected in public health and education. The national healthcare system, while acknowledging menopause, lacks specific treatment frameworks beyond preventative care (33). Similarly, the education system, despite increasing health-related content, fails to explicitly address women's health issues (34), such as menopause, in official curricula (35, 36). Although legislation such as Ley Orgánica 1/2023 (37) addresses menstrual health, it overlooks the specific needs of women in menopausal transition and postmenopause. However, regional initiatives, such as the Pla Integral d'Equitat Menstrual i Climateri 2023–2025 (38) in Catalonia, demonstrate a promising approach that aims to improve healthcare, social visibility, and equity in women's health, and recognize menopause as a key stage requiring sensitive and adequate support.

In this context of limited understanding and inconsistent support, this article aims to identify and analyze proposals for action that public organizations in Spain could undertake to improve the management of and transition through menopause. On the basis of the initiatives identified through women's narratives, this study seeks to inform future governmental policies and practices adapted to the unique sociocultural and economic conditions of each region, potentially stimulating reflection and debate on menopause policies both within Spain and internationally.

## 2 Materials and methods

To address our research objectives and enable an in-depth understanding and interpretation of the topic, we opted for a phenomenological qualitative design. This approach allowed us to explore the lived experiences of women during menopause, with a focus on how they perceive and make sense of this stage of life.

### 2.1 Research rigor and reflexivity

This study was conducted by a single researcher with a background in public health and experience in qualitative research methods, including phenomenology. To ensure research rigor and address potential biases inherent in this design, several strategies were implemented. Specifically, a research diary was kept to document methodological decisions, reflect on the research process, and identify and challenge any personal biases or assumptions that may have influenced the study. Additionally, two researchers experienced in qualitative methodology conducted a peer review of the study design, interview guide, and data analysis process, which helped to enhance the rigor and trustworthiness of the study. Finally, to ensure accurate interpretation of the participants' experiences, the findings were validated with the participants themselves. They were provided with a summary of the results and the opportunity to provide feedback and verify that their experiences were accurately represented.

### 2.2 Geographical scope

As stated, this study was conducted in Spain, a particularly suitable environment for the analysis of menopause due to the specific features of its healthcare system and its progressive gender equality policies (33, 37, 38). Legislation addressing gender violence, promoting work‒life balance, and guaranteeing equal pay, together with the significant number of women in the menopausal stage, constitute an enriching context for this research. However, when interpreting the results, it is essential to consider the particularities of this social and cultural context to assess its applicability in other settings.

### 2.3 Participants

The study recruited participants through Facebook and Instagram advertisements, along with partnerships with organizations supporting menopausal women. Purposive sampling was used to include cisgender women who met specific criteria: (a) having experienced symptomatic menopause, defined as the physiological cessation of menstruation accompanied by typical menopausal symptoms, within the past five years, which reduces recall bias and allows for an in-depth exploration of the impact of symptoms on quality of life and work; (b) being postmenopausal, defined as a period of 12 months or more without menstruation; and (c) being actively employed, to analyze the influence of menopause on work experience, well-being, and career trajectory. This approach sought to ensure diversity in life stages, family structures, educational levels, professional sectors, and job roles.

As shown in Table 1, the study sample consists of 20 women, mostly Spanish, aged between 46 and 60 years, with varying educational levels, from basic studies to PhDs. Most of the participants are in a relationship, and many of them have children. Professionally, they are employed in sectors such as public administration, technology, education, and health, holding roles ranging from managerial to executive positions.

**Table 1:** Interviewee profiles.

ID	Current age	Menopause age	Nationality	Current residence	Educational level	Relationship status	Family status	Professional sector	Job occupation
1	50	49	Spain	Barcelona	Elementary school	In a relationship	Two children, aged 16 and 19	Public administration	Non-managerial
2	49	48	Spain	Sevilla	High school	In a relationship	Two children, aged 14 and 19	Administration and management	Manager
3	52	50	Spain	Madrid	Bachelor's degree	Divorced	Two children, aged 7 and 9	Technology	Principal
4	53	48	Spain	Barcelona	Master's degree	Single	No children	Education	Manager
5	57	52	Spain	Barcelona	Bachelor's degree	Divorced	Two children, aged 11 and 13	Technology	Director
6	52	50	Spain	Zaragoza	High school	In a relationship	Three children, aged 17, 19 and 21	Social	Director
7	46	44	Spain	Pamplona	Elementary school	In a relationship	No children	Education	Non-managerial
8	54	51	Spain	Barcelona	Elementary school	In a relationship	Two children, aged 16 and 21	Administration and management	Non-managerial
9	54	51	Spain	Bilbao	High school	In a relationship	Two children, aged 21 and 29	Public administration	Manager
10	55	50	Spain	Oviedo	High school	In a relationship	In charge of mother and brother	Public administration	Non-managerial
11	57	54	Spain	Barcelona	Master's degree	In a relationship	Two children, aged 30 and 32	Social economy	Director
12	60	55	Morocco	Murcia	Middle school	In a relationship	Three children, aged 21, 22 and 25	Environmental services	Non-managerial
13	57	55	Spain	Madrid	Bachelor's degree	Single	No children	Services to companies	Manager
14	58	53	Venezuela	Barcelona	Bachelor's degree	In a relationship	Four children, aged 28, 32, 35 and 38	Environmental services	Director
15	54	52	Spain	Lugo	Middle school	Widowed	Two children, aged 30 and 32	Healthcare	Non-managerial
16	47	44	Spain	Girona	Doctoral degree	In a relationship	No children	Logistics	Manager
17	53	51	Spain	Madrid	Bachelor's degree	Divorced	One child, aged 17	Services to companies	Director
18	58	53	Spain	Barcelona	Doctoral degree	Widowed	One child, aged 30	Logistics	Director
19	49	47	Spain	Valencia	High school	In a relationship	Two children, aged 14 and 16	Healthcare	Manager
20	57	54	Spain	Tarragona	Bachelor's degree	In a relationship	Two children, aged 21 and 24	Services to companies	Director

### 2.4 Instruments and procedures

We gathered data through semistructured interviews via a theme-centered script based on previous research (17) and aligned it with the specific objectives of this study. The script included open-ended questions focusing on five main aspects: personal experiences during the menopausal transition and postmenopause; the quality of medical care; institutional and community support; the impact of existing public policies; and cross-cutting proposals for improvement. The interview questions were refined on the basis of feedback from initial interviews to ensure clarity and depth.

Eligible participants were contacted by email and provided with an informed consent document, study information, and a link to schedule an online or in-person interview, according to their preference. Online interviews were conducted via the Zoom platform, which offers a secure and encrypted environment to protect participant privacy. Video call options were used to facilitate interaction and connection between the researcher and participants. In-person interviews were conducted in a private meeting room at the Universitat Oberta de Catalunya (UOC), which provided a quiet and comfortable space for participants.

The interviews were conducted from April to June 2024, 12 of which were online and 8 were in person. The interviews ranged from 40 to 60 min in duration and were audio-recorded to ensure accurate verbatim transcription. Data saturation was reached when new interviews did not provide significant new information (39, 40).

### 2.5 Data analysis

To identify common themes and patterns in the interviews, we conducted an inductive content analysis (41). This analytical procedure took place in three consecutive phases. First, after careful reading, a preanalysis was performed to familiarize ourselves with the data and identify relevant concepts at an early stage. In this initial phase, notes were taken, and summaries were drawn up to organize the first impressions and reflections on the data. Next, the data were segmented and coded via the analytical software Atlas.ti (version 8.4.21.0), which was chosen for its efficiency in handling large volumes of data and the features it offers for systematic coding. This process allowed us to identify the following ten codes that reflect the key concepts and ideas expressed by the participants: information campaigns, equipping public spaces for menopause support activities, offering specialist services and advice in health centers, appointing a menopausal ambassador and highlighting role models, including menopause in the school curriculum, training educators and social workers, ensuring that healthcare professionals receive quality training, including menopause in equal rights law, legislating menopause in the workplace, and funding menopause research. Finally, in the third phase, we grouped the codes into three categories that emerged through the constant comparison method, which compares similar and different facts to enrich and explain the existing categories more effectively. The three categories that emerged from this analysis were proposals for accompaniment and social awareness, educational and training proposals, and political and regulatory proposals.

### 2.6 Ethical considerations

This study was approved by the Ethics Committee of the Universitat Oberta de Catalunya (UOC, Exp.: CE24-PR14). Before the interviews, we explained the goal of the research to the participants and gave them an information sheet so that they could refer to that information whenever they wanted. Throughout the study, we implemented measures to ensure the participants' rights and well-being, voluntary participation, informed decision-making, anonymity, and safeguarding of any confidential information. These practices are in line with the legal regulations on data confidentiality in the Spanish Data Protection Act, Organic Law 3/2018 of 5 December 2018.

## 3 Results

**Figure 1:**
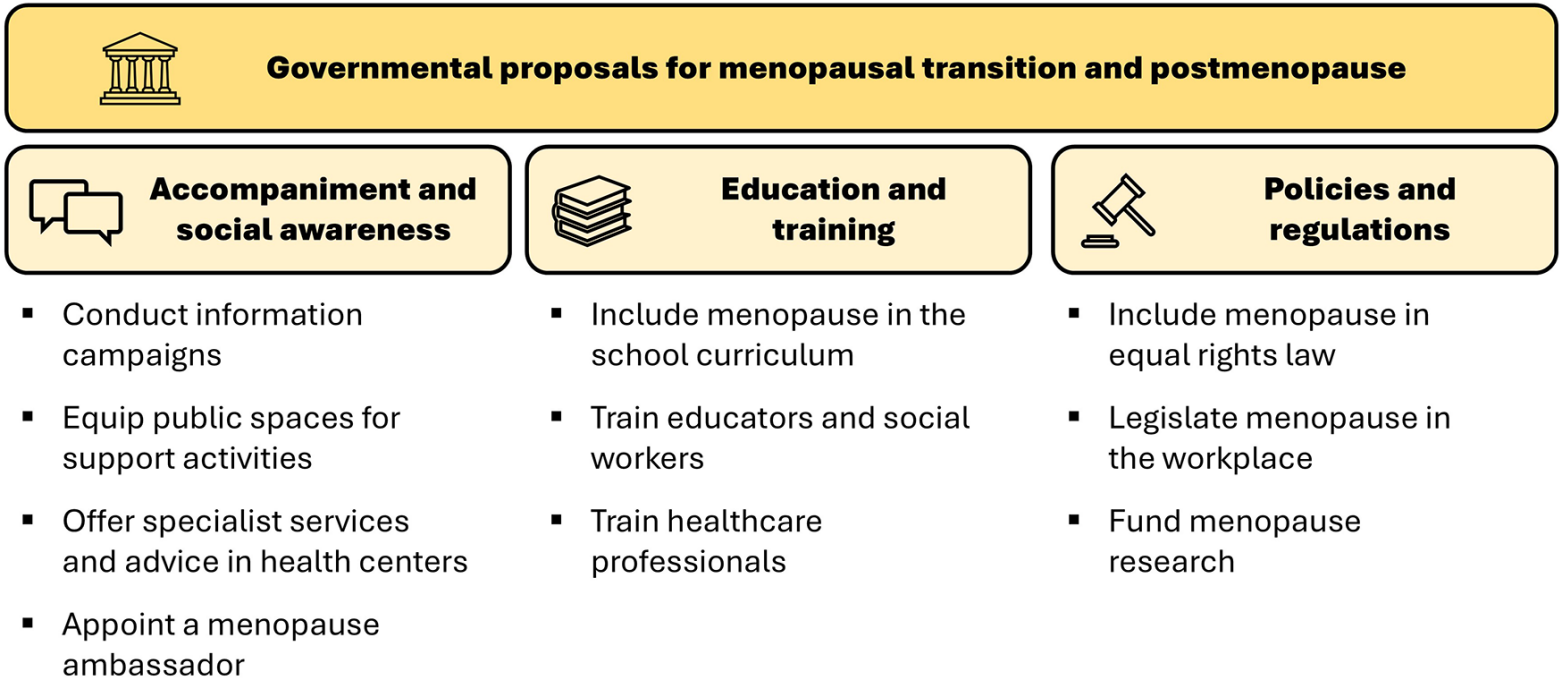
Categories and specific measures of government action.

As shown in Figure 1, the stories of the interviewees indicate three large categories of government action comprising a set of specific actions, which are detailed below.

### 3.1 Proposals for accompaniment and social awareness

The first category includes four specific measures to increase the public visibility of menopause, raise awareness of its effects, and develop strategies for accompaniment, normalization and acceptance.

#### 3.1.1 Information campaigns

The narratives highlight the importance of providing public information about menopause to increase awareness, normalize it, and provide support to those experiencing it. This is especially relevant in disadvantaged or low-income populations, where the scarcity of information can exacerbate problems of understanding or lack of support. According to the interviewees, these campaigns could include sending letters with information to people approaching this stage of life, inviting them to use primary care services, and providing a kit with specific products to improve their experience of menopausal transition and postmenopause:

"Governments must make information available and offer personalized education and support, free of charge, to people who need it. In the end, these campaigns could make menopause easier to understand and more natural, and this would be even more important in vulnerable areas, where it can be more difficult to obtain this information.” (Interview 10)

“They could make calls or send information letters offering support and offer kits that help with the most common symptoms, such as hot flashes, night sweats, dryness… That could be done cheaply, couldn’t it? With moisturizing products, herbal teas, or leaflets with advice on nutrition, sleep hygiene, self-care, exercise… everything that helps you look after your health.” (Interview 7)

The proposed actions not only have the potential to remedy the lack of awareness about menopause, which often leads to isolation, lack of support, and shame, but also, as some women have expressed, can transform the way this life stage is seen:

“There’s lots of stuff about happy-happy subjects like maternity, but menopause is in the uncomfortable, hushed-up, taboo place that mental health used to be in, and now it’s so normalized… Governments have a lot to do if they want to help us: talk, talk, talk about it! A lot of education, and then people talking about it to each other will do the rest.” (Interview 1)

*“We live in a sexist world, men dominate, they’re in charge, they create the discourse and make the laws. For this to change, we need knowledge, more awareness in society, and to create new directions and meanings for everything which has to do with women, especially regarding menopause, which isn*’*t a drama, just a different stage with consequences and situations which can be controlled as we go.”* (Interview 8)

#### 3.1.2 Equipping public spaces for menopause support activities

The interviewees repeatedly mentioned the opportunity to use public spaces, owing to their proximity and welcoming atmosphere, for information and social support actions, such as meetings or “menopause coffees”, training groups, or neighborhood support groups. More specifically, they believe that this new use of public spaces could facilitate sharing experiences and promote open, friendly, and supportive dialog on the topic:

“It would be a good idea to have round tables and ‘menopause café’-type meetings in public spaces, where people can share personal experiences.” (Interview 4)

“[..] there are many underused municipal facilities where there could be campaigns or support groups where women could share our experiences or get information.” (Interview 14)

In this sense, the opportunity to use these spaces is based on their public ownership and the availability of restrooms and drinking water fountains for those who may need them:

“I have always thought that these spaces aren’t used enough, and as we have well-equipped sports centers with toilets and running water, why not give them a second use with women’s healthcare activities? I’m sure it would be very popular!” (Interview 20)

#### 3.1.3 Offering specialist services and advice in health centers

According to the interviewees, health centers should provide accessible and inclusive healthcare services and professional advice for people in menopause, including emotional and psychological support programs, discussion groups, workshops on healthy habits, or individualized guidance:

“It would be great if health centers offered recommendations on nutrition, exercise, healthy habits, emotional and psychological support…” (Interview 19)

“It would be truly good to have more resources in the health centers… How about discussion and support groups or practical workshops? That would be useful.” (Interview 14)

As they affirm, the presence of specialized professionals in these spaces can be very helpful for educating people and improving their personal management of this life stage with accurate information and practical advice:

“Primary care centers could organize women’s support groups, supervised by a nurse, a psychologist or a doctor, where we could share our experiences [..] just talking about it would make people feel better, because that’s when you realize you don’t actually have a problem.” (Interview 6)

#### 3.1.4 Appointing a menopausal ambassador and highlighting role models

For some interviewees, the need to raise the profile of people going through menopause and give them a public voice could take the form of a “menopause ambassador”. This representative would encourage understanding of and social support for this life stage, ensuring that those experiencing it have access to the services and resources they need to manage their symptoms and improve their well-being. As the main link between people in menopausal transition and postmenopause and government healthcare, well-being and education services, the “menopause ambassador” must have holistic, multidisciplinary expertise on this life transition:

“[..] we need someone who represents us, someone who makes sure we get the support and resources we need during menopause. I’ve read that some other countries already have this type of public figure, and it should be applicable everywhere…” (Interview 16)

Similarly, it is crucial to increase the representation of this collective in the media and empower all of them to share experiences, provide resources and offer advice on menopause through various informative channels to eliminate the social invisibility associated with this stage:

"[..] we live in a society so obsessed with youth and beauty that when we get to this age, we become invisible. If you watch TV, there are hardly any women who represent us, and that's also the responsibility of governments, who should be making sure there is equal representation of all women in different stages of life.” (Interview 11)

“[..] they should do things thinking of all the outlets because if you only do TV adverts, many people won’t have that information because they don’t watch TV. [..] we need all the formats (TV, online, podcasts, apps…), forms (written, narrated, visual…) and role models (in healthcare, in society, in show business, on Instagram.. so there can be models for everyone.” (Interview 15)

### 3.2 Educational and training proposals

This second category consists of three specific proposals for educating and training people in schools and places of work about menopause, contributing to promoting more equitable and well-informed societies.

#### 3.2.1 Including menopause in the school curriculum

The narratives converge on the relevance of including menopause in the school curriculum to foster an early, complete, and naturalized understanding of the female life cycle. In fact, this early knowledge could help demystify the changes of this stage and promote an attitude of respect and empathy throughout society:

“Children should be taught and have information shared with them at school, from an early age, but not prettied-up, it should be clear, so they are all aware of it [..] The female life cycle should be a basic subject so they understand about periods, pregnancy, and menopause, and tell them straight: ‘If you’re a woman this is going to happen whether you like it or not, so you might as well get used to the idea..’” (Interview 9)

Moreover, they believe that this educational approach should promote a clear and comprehensive understanding of this life cycle process, which not only helps ensure that future generations are better informed and supported in their transitions but also contributes to a better understanding of the emotional and physical needs associated with menopause:

"And education about menopause should not just be about the biological part, but include the psychological, social, everything involved. It's the only way our children will be ready to be an active part of this process.” (Interview 12)

#### 3.2.2 Training educators and social workers

To ensure that information on menopausal transition and postmenopause is adequate, transmitted with respect, and understood in a positive and natural way, the interviewees pointed to the need to provide specific training to education professionals, including the biological, emotional, and psychosocial aspects of this stage of life:

“If menopause is added to the school curriculum, I think the most important thing is for the teachers to be well prepared, with appropriate, accurate and understandable training, and for the subject to be treated with the sensitivity and respect it requires, with a good overview…” (Interview 18)

In this sense, social service professionals need to receive training in emotional support techniques and have access to updated information on existing public resources, thus optimizing their ability to support and guide people through menopause:

“They [social services professionals] should also offer emotional support and tell us about the resources and services available in different places to improve our experience, right?” (Interview 10)

#### 3.2.3 Ensuring that healthcare professionals receive quality training

Given that menopause affects half of the world's population, a good proportion of women consider it essential that medical schools guarantee compulsory training to ensure that professionals can provide quality specialist healthcare:

“Governments should ensure that they receive mandatory training that enables them to diagnose, guide and support in a comprehensive and multidisciplinary way. In addition, not just that, they should also guarantee working with an individual approach, understanding that each of us can have different needs..” (Interview 7)

Additionally, the introduction of a specific consultation point in health centers, staffed by these specialists, would mean that people going through menopause could be properly examined and receive personalized advice:

“Having a well-trained female professional specializing in menopause in your health center would be like having an ally in this battle, like opening a door of help and understanding at a time when you feel lost and ignored, sometimes badly advised or even treated like a number…” (Interview 15)

### 3.3 Political and regulatory proposals

This third category includes three specific proposals aimed at establishing and supervising the implementation of policies and legal regulations on menopause in public and private spaces.

#### 3.3.1 Including menopause in equal rights law

According to the interviewees, including menopause in gender equality legislation means recognizing this life stage as being legally relevant to society and ensuring the implementation of measures to protect and improve the quality of life and the social and labor rights of the people experiencing it:

“[..] Developing flexible and equitable policies is urgent!! However, working from macro to micro, you know? And for a line of government action to result in a line of social action, in organization… you get me?” (Interview 4)

In addition to these regulations, they consider it essential to ensure the provision of adequate public facilities to meet the specific needs of menopausal transition and postmenopause. This includes equipping public spaces with toilets with adequate hygienic conditions that are accessible to those who need them and ensuring the availability of drinking water fountains:

“There should be public toilets all over town, because if you’re out for a walk, which is one of the most recommended ways to avoid weight gain, and you need to go, or you’re bleeding a lot, you can use them and change your clothes or freshen up…” (Interview 12).

#### 3.3.2 Legislating menopause in the workplace

Governments should use legislation to implement action plans to prevent inequality and reduce the gender pay gap in organizations. However, women note that the first step should be to provide resources and support that facilitate the adoption of inclusive and menopause-sensitive policies and practices, such as flexible schedules, remote work, “menopause leave,” or shorter working hours.

“[Governments] must wake up and require companies to create real plans to avoid inequality and manage this life stage optimally, but first they have to help them, train them, give them resources… and once they’ve done that and given them enough time, they can start making demands, right?” (Interview 2)

“[…] organizations should be proactive and not reactive, letting things happen. They should predict what these women need and look for solutions: Is it starting and ending work within a range of times? Or changing shifts or work locations? Or enhancing telework or hybrid work? Or does one offer ‘menopause leave’? They should study whether these things can be done.” (Interview 12)

Furthermore, they believe that these flexibility measures should be complemented with regular monitoring to ensure their correct implementation, along with financial incentives to encourage their adoption in the workplace.

“This can’t end up on a piece of paper filed and forgotten; the government has to monitor it to verify that their action proposals are implemented, right?” (Interview 11)

“[…] and if companies step up and expand and incentivize flexible work, or hybrid or telework, they should be compensated…” (Interview 3)

#### 3.3.3 Funding menopause research

Lastly, from the interviewees' narratives, it is concluded that promoting and funding research on menopause is a priority, especially regarding treatments and therapies to mitigate symptoms and prevent potential long-term health problems:

“[..] more money needs to be invested in research and development of treatments that improve our quality of life. Otherwise, we women will continue to suffer from symptoms that could undoubtedly be relieved with more appropriate therapies.” (Interview 13)

For this purpose, more scientific evidence is needed to enable approaching this life transition in a less overmedicated and more natural way, including changing habits and lifestyles:

“We need more research into nonpharmacological options for menopause, and I think this could be done easily with cross-department working committees from different government departments…” (Interview 17)

“[..] my experience changed when I explored the benefits of natural methods: exercise, a healthy diet, changing my lifestyle.. However, I didn’t learn this from a doctor, but by going to more natural types of therapies. When doctors find a balance with this other branch, I think that’s when we’ll truly improve the management of this life stage, so we need to support research to back it up.” (Interview 1)

## 4 Discussion

The results of our research offer a valuable contribution to understanding the needs and experiences of women during the menopausal transition and postmenopause in Spain and to identifying proposals for action that can guide the development of more effective public policies and workplace practices. With the potential to adapt to the social and cultural characteristics of each country, these proposals are based on three key needs: (a) empowering proposals for guidance and social awareness; (b) launching educational proposals; and (c) implementing policy and regulatory proposals.

### 4.1 Proposals for accompaniment and social awareness

Often, silence and a lack of information and basic knowledge intensify myths, stigmas, and taboos around this life stage (4, 12). For this reason, public organizations need to promote information campaigns that can visualize, normalize and accompany this stage, thus helping reduce the embarrassment that often accompanies it. These campaigns should consider the specific cultural and linguistic diversity in Spain to ensure inclusivity and accessibility for all women. Similarly, maximizing the use of certain public spaces for menopause-related activities could contribute to fostering social activism and a sense of sisterhood among this collective, essential elements for reducing the stigma associated with this life stage (42, 43). In line with research on menstrual justice (9), these measures seek to challenge the cultural silence and shame associated with menopause, creating spaces for open dialog and support (5).

While introducing specialist services in health centers represents a significant step toward integrated menopause support, the challenge is to ensure a multidisciplinary approach among healthcare professionals and universal accessibility for the population as a whole, thus facilitating effective and empathetic management of their specific needs (44, 45). In the Spanish context, where healthcare is decentralized (33), ensuring consistent quality and access to specialized menopausal care across different regions is crucial. This could involve establishing clear guidelines and protocols for menopause management within the national healthcare system, as well as investing in training and resources for healthcare professionals. Finally, taking successful initiatives in other countries as points of reference, appointing a menopausal ambassador, and highlighting role models with exhaustive knowledge of this life stage could ensure fairer and more equitable access to treatments, information and support, as well as more correct representation in the media.

In line with other studies (3, 12, 13), it is crucial that governments raise awareness of and normalize the menopausal transition and postmenopause, and encourage open conversation among the population, similar to other life stages, such as adolescence or pregnancy (13). Beyond this, it is necessary to question and redefine the social meanings traditionally associated with this stage to foster a more liberating discourse, free of the patriarchal connotations previously related to women's reproductive and nonreproductive cycles, especially menopause (34, 36).

However, deconstructing the current imaginary and creating a new one is not a simple task; it requires wide-ranging education work and dissemination by public bodies, as well as considerable efforts by individuals, to gradually progress toward maximum understanding and acceptance of this life stage. In brief, all these actions share the goal of driving a paradigm shift to make Spanish society more inclusive and empathetic in relation to menopause and ensure that the representation of those experiencing it is fairer and more accurate.

### 4.2 Educational and training proposals

As a physiological stage in reproductive health, it is essential that schools teach students about menopausal transition and postmenopause. In line with the conclusions of other studies, including this stage and all its dimensions (physical, biological, emotional, and psychosocial) in educational material on sexual and reproductive health can foster an earlier, more empathetic and unprejudiced understanding of women's health (1, 34). This is particularly important in Spain, where comprehensive sexual education has not yet been universally implemented in schools (35, 36). It is also necessary for the different professionals involved in dealing with menopause (such as healthcare workers, educators, and social workers) to obtain specific, holistic training, facilitating a more effective, sensitive, and personalized approach (34). In this way, they can promote a better understanding and supportive approach to these transitions, including advice on the available services and support that may be needed (12).

These results are in line with various studies that more specifically highlight the need for at least one specialist doctor or healthcare professional in each health center, or for funding, and requiring the participation of these professionals in specific training programs to ensure competent and up-to-date management of their needs (36, 37). In the Spanish context, this could involve strengthening the training of primary care doctors and nurses in menopause management, as they are often the first point of contact for women seeking information and support. In short, these actions are intended to educate and ensure comprehensive support for menopausal transition and postmenopause, contributing to breaking free from the taboos and stigmas around it.

### 4.3 Political and regulatory proposals

The recognition and inclusion of menopause in the legal framework protects the labor and social rights of people experiencing it. In accordance with previous research, gender equality policies, which include specific provisions on menopause, can significantly reduce discrimination and improve the well-being of those experiencing it (15, 16, 46). More specifically, the implementation of regulations such as the availability of public toilets and drinking water fountains can drive the creation of healthier and more sustainable environments for the population as a whole. This aligns with the growing interest in environmental health and sustainability in Spain.

At the organizational level, it is crucial that, within this legal framework, the Spanish government advises organizations to adopt inclusive and menopause-sensitive policies and practices (such as the right to take time off work for menopause-related discomfort or the provision of benefits similar to those for maternity). After this, they must implement specific action plans, with appropriate monitoring of their effectiveness, and could provide subsidies to reward best practices in managing the menopausal transition and postmenopause. Finally, given the knowledge gaps in research on this life stage, it is necessary to fund and support studies related to treatments, therapies, and lifestyles that can mitigate symptoms and prevent potential long-term health problems (8). In short, these actions are intended to regulate the management of menopause and contribute to greater equity and social justice in relation to it. Beyond the Spanish context, it should be noted that some of these measures are already in place in certain countries, but their application is still mostly limited to contexts and regions with high awareness of menopause (37, 38).

## 5 Conclusion

This phenomenological study has identified and analyzed proposals for action to improve the management of and transition through menopause in Spain, a stage traditionally neglected in public policies. While Spanish legislation has advanced regarding women's health in different life stages in recent years, there is still a need to delve deeper into specific policies that allow for an improved experience and social conception of menopause. On the basis of women's narratives, this study identified needs and concrete actions that could help breakdown taboos, increase visibility, eliminate discrimination, and raise awareness of the impact of menopause on quality of life.

The findings of this study, which focused on the experiences of women in Spain, provide a solid basis for developing public policies and workplace practices that respond to their needs. As evidenced by its results, collaboration between governments, society, and organizations is crucial to achieve real change that increases understanding and support, and eradicates discrimination and the risk of social exclusion. Future studies should explore the adaptation of these proposals to different socioeconomic and cultural realities, considering factors such as age, ethnicity, sexual orientation, and socioeconomic status, to offer more personalized and inclusive solutions that benefit all people experiencing menopause.

## Data Availability

The raw data supporting the conclusions of this article will be made available by the authors, without undue reservation.
